# Characteristics of patients with Coronavirus Disease 2019 (COVID-19) and seasonal influenza at time of hospital admission: a single center comparative study

**DOI:** 10.1186/s12879-021-05957-4

**Published:** 2021-03-17

**Authors:** Pablo Sieber, Domenica Flury, Sabine Güsewell, Werner C. Albrich, Katia Boggian, Céline Gardiol, Matthias Schlegel, Robert Sieber, Pietro Vernazza, Philipp Kohler

**Affiliations:** 1 Division of Infectious Diseases and Hospital Epidemiology, Cantonal Hospital St. Gallen, Rorschacher Strasse 95, 9007 St. Gallen, Switzerland; 2 Clinical Trial Unit, Cantonal Hospital St. Gallen, St. Gallen, Switzerland; 3 Federal Office of Public Health, Berne, Switzerland; 4 Emergency Department, Cantonal Hospital St. Gallen, St. Gallen, Switzerland

**Keywords:** COVID-19, SARS-CoV-2, Influenza, Differences, Comparative, Classification tree

## Abstract

**Background:**

In the future, co-circulation of severe acute respiratory syndrome coronavirus 2 (SARS-CoV-2) and influenza viruses A/B is likely. From a clinical point of view, differentiation of the two disease entities is crucial for patient management. We therefore aim to detect clinical differences between Coronavirus Disease 2019 (COVID-19) and seasonal influenza patients at time of hospital admission.

**Methods:**

In this single-center observational study, we included all consecutive patients hospitalized for COVID-19 or influenza between November 2019 and May 2020. Data were extracted from a nationwide surveillance program and from electronic health records. COVID-19 and influenza patients were compared in terms of baseline characteristics, clinical presentation and outcome. We used recursive partitioning to generate a classification tree to discriminate COVID-19 from influenza patients.

**Results:**

We included 96 COVID-19 and 96 influenza patients. Median age was 68 vs. 70 years (*p* = 0.90), 72% vs. 56% (*p* = 0.024) were males, and median Charlson Comorbidity Index (CCI) was 1 vs. 2 (*p* = 0.027) in COVID-19 and influenza patients, respectively. Time from symptom onset to hospital admission was longer for COVID-19 (median 7 days, IQR 3–10) than for influenza patients (median 3 days, IQR 2–5, *p* < 0.001). Other variables favoring a diagnosis of COVID-19 in the classification tree were higher systolic blood pressure, lack of productive sputum, and lack of headache. The tree classified 86/192 patients (45%) into two subsets with ≥80% of patients having influenza or COVID-19, respectively. In-hospital mortality was higher for COVID-19 patients (16% vs. 5%, *p* = 0.018).

**Conclusion:**

Discriminating COVID-19 from influenza patients based on clinical presentation is challenging. Time from symptom onset to hospital admission is considerably longer in COVID-19 than in influenza patients and showed the strongest discriminatory power in our classification tree. Although they had fewer comorbidities, in-hospital mortality was higher for COVID-19 patients.

**Supplementary Information:**

The online version contains supplementary material available at 10.1186/s12879-021-05957-4.

## Background

Coronavirus Disease 2019 (COVID-19) is currently having a major impact on global health with over hundred million confirmed cases and over two million deaths by February 2021 [1]. On the other hand, annual influenza epidemics causes 300,000 to 500,000 deaths globally [2, 3]. In the future, co-circulation of severe acute respiratory syndrome coronavirus 2 (SARS-CoV-2), causing COVID-19, and influenza viruses A/B, causing seasonal influenza, is likely.

From a clinical point of view, differentiation of the two disease entities is crucial for patient management. For instance, the administration of steroids has been advocated for patients with COVID-19 [4]; yet, steroids are contraindicated for influenza patients [5]. Furthermore, diagnostic tests for both COVID-19 and influenza are not always accurate [6, 7]. Also, these tests are costly and often not readily available [8]. Clinical presentations of COVID-19 and influenza share many similarities, although some differences have been reported [9–16]. However, many of these previous studies had a rather small sample size and they often focused on specific aspects such as stroke or acute respiratory distress syndrome (ARDS) [10, 11, 13, 16]. Also, in one study COVID-19 and influenza patients were recruited from different hospitals, introducing selection bias; similarly, in another study COVID-19 patients were compared to influenza patients from previous seasons, which also limits the comparability of groups [11, 12].

In this single-center study, we compared baseline characteristics and clinical findings between COVID-19 and influenza patients upon presentation to the emergency department. These results could hopefully aid clinicians in the early management of patients presenting with acute respiratory illness during influenza season, particularly in case of inconclusive test results or lack of these.

## Methods

### Study design, population and setting

In this single center observational study, we included patients presenting to the emergency department of our 800-bed tertiary hospital in Eastern Switzerland between 25th of November 2019 and 7th of May 2020. For diagnosis of either COVID-19 or influenza A/B, a positive laboratory test results was required. Between November 1st 2019 and April 1st 2020, patients presenting with acute respiratory illness (ARI) routinely underwent testing for influenza A and B (Alere or multiplex PCR); starting from March 1st 2020, a SARS-CoV-2 PCR (realtime-PCR or GeneXpert) was routinely performed on nasopharyngeal swabs from ARI patients. In case of an initial negative test for SARS-CoV-2 or influenza, but high clinical suspicion, a second PCR - either again from the nasopharynx or from specimens of the lower respiratory tract - was performed. Only patients admitted to the general ward or the intensive care unit (ICU), with a minimum stay of 24 h, were included. Criteria for hospital or ICU admission for COVID-19 and influenza were the same during the study period and were mainly based on clinical judgment. Patient triage due to limitations in hospital or ICU bed capacities was not necessary during the entire study period.

Exclusion criteria were age younger than 16 years, nosocomial infections (defined as positive laboratory test after 5 days of hospitalization for COVID-19 or after 3 days for influenza), rejection of the general consent, and transfers from other hospitals (if they had been admitted more than 24 h before transfer). The study was approved by the Ethics Committee of Eastern Switzerland (project ID: 2020–01347), located in St. Gallen, Switzerland.

### Data sources

COVID-19 and influenza patients were prospectively registered in two national surveillance studies [17] from where patient identities as well as baseline variables were extracted. In addition, we used a study specific case report form (CRF) and collected details on comorbidities, clinical presentation (symptoms, vital signs) and laboratory findings from the electronic patient records. Also, outcome variables including ICU admission, length of stay (LOS) for those discharged alive (both hospital and ICU LOS), antibiotic and steroid use, and in-hospital death were collected. We calculated the following scores for patients with available data: Charlson Comorbidity Index (CCI) [18]; CURB-65 score [19]; and Quick-SOFA score [20].

### Statistical analysis

For descriptive analysis, we computed numbers and percentages for categorical data and medians with interquartile ranges (IQR) for numeric data. To compare the two groups, we used the Pearson’s chi-squared test or the Fisher’s exact test for categorical data, as appropriate. For continuous data, we used the Mann-Whitney U test. A two-sided *p*-value of < 0.05 was considered statistically significant. These statistical analyses were performed using SPSS from IBM (version 20).

We further used recursive partitioning to classify patients into subsets with distinct proportions of COVID-19 cases based on their characteristics at the time of hospital admission. Variables were included if data were available from at least 80 patients per group. Recursive partitioning is a non-parametric procedure that successively splits a data set into subsets (called “nodes”) by defining cutpoints on predictor variables. At each step of the procedure, one predictor and one cutpoint are automatically selected to maximize the difference in outcome distribution (here: COVID-19 proportion) between the two resulting subsets. These two subsets can be further subdivided using different predictors. We allowed splitting to continue as long as a subdivision was significant at the 5% level (without adjustment for multiple testing) and as long as the subset to split included at least 70 patients. This analysis was performed with the package party in the software R, version 4.0.2 (R Foundation for Statistical Computing, Vienna, 2020). A classification (i.e. a combination of characteristics) was considered clinically helpful if the observed proportion of either COVID-19 or influenza cases in the resulting patient subset was at least 80%.

## Results

### Study population and local epidemic curves

**Fig. 1:**
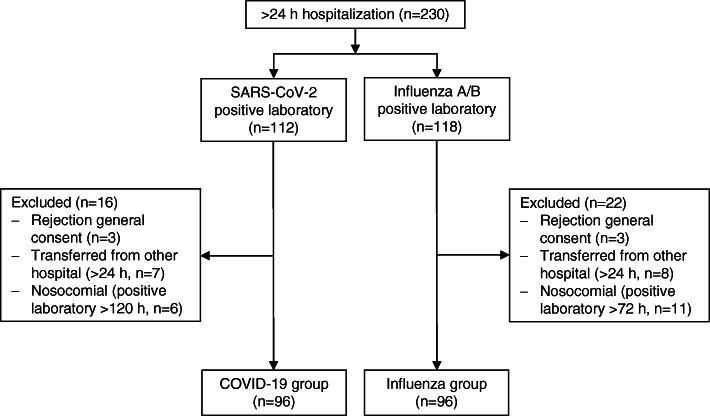
Inclusion criteria. Abbreviations: SARS-CoV-2, Severe acute respiratory syndrome corona virus 2; h, hour

**Fig. 2:**
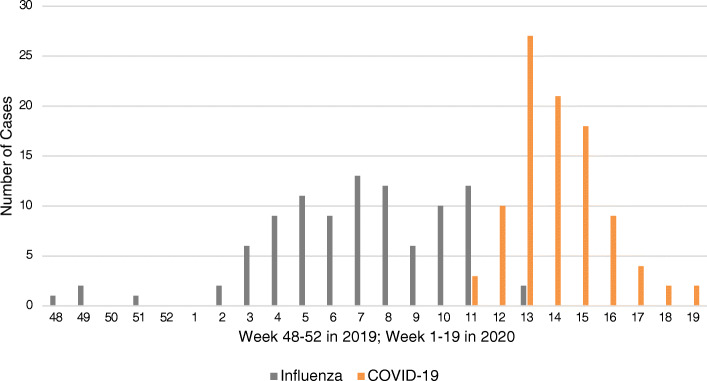
Hospital admission timeline

Between November 25th 2019 and May 7th 2020, 112 patients were hospitalized with COVID-19, and 118 with seasonal influenza. Thereof, 96 COVID-19 and 96 influenza patients were included in the study (Fig. 1). Whereas patients with influenza were hospitalized between November 25th and March 26th (with a peak in February 2020), the first COVID-19 patient was admitted on March 10th; a peak of hospitalized COVID-19 was reached by the end of March 2020 (Fig. 2).

### Baseline characteristics

Patients had a median age of 68 years (IQR 57–81) for COVID-19 and 70 (IQR 57–80) years for influenza (*p* = 0.90); males were more common among COVID-19 patients (72% vs 56%, *p* = 0.024); COVID-19 patients were more often transferred from long-term care or rehabilitation clinics than influenza patients (15% vs 5%, *p* = 0.030). The proportion of active smokers was lower in COVID-19 compared to influenza patients (4% vs 13%, *p* = 0.033). COVID-19 patients had less comorbidities compared to influenza patients (median CCI 1 vs 2, *p* = 0.027), which was mostly due to less chronic renal disease (21% vs 35%, *p* = 0.025), less oncological disease (8% vs 26% *p* = 0.001), and less chronic respiratory disease (10% vs 21%, *p* = 0.047) (Table 1).

**Table 1:** Demographics of COVID-19 vs Influenza patients

Characteristics	COVID-19 (*n* = 96)		Influenza (*n* = 96)		*p*-value
Age, median (IQR)	68	(57–81)	70	(57–80)	0.901
Male	69	(71.9%)	54	(56.3%)	0.024*
Body mass index, median (IQR)^a^	27	(24.5–30.7)	26	(23.4–29.5)	0.204
Pregnancy	1	(1.0%)	5	(5.2%)	0.211
Smoker status					
Current	4	(4.2%)	12	(12.5%)	0.033*
Former	27	(28.1%)	21	(21.9%)	
Never	33	(34.4%)	21	(21.9%)	
Unknown	32	(33.3%)	43	(44.8%)	
Nationality					
Swiss	79	(82.3%)	74	(77.1%)	0.370
Non-Swiss	17	(17.7%)	22	(22.9%)	
Transfer from					
Home	82	(85.4%)	91	(94.8%)	0.030*
Health care institution^b^	14	(14.6%)	5	(5.2%)	
Comorbidities					
Charlson Comorbidity Index, median (IQR)	1	(0–2)	2	(0–3)	0.027*
Hypertension	53	(55.2%)	48	(50.0%)	0.470
Chronic cardiovascular disease	40	(41.7%)	50	(52.1%)	0.148
Chronic respiratory disease	10	(10.4%)	20	(20.8%)	0.047*
Chronic renal disease	20	(20.8%)	34	(35.4%)	0.025*
Chronic liver disease	2	(2.1%)	9	(9.4%)	0.030*
Diabetes	28	(29.2%)	23	(24.0%)	0.414
Obesity^c^	21	(22.6%)	19	(24.7%)	0.749
Oncological pathology	8	(8.3%)	25	(26.0%)	0.001*
Osteoporosis	6	(6.3%)	6	(6.3%)	1.000
Rheumatologic disease, immunosuppressed	5	(5.2%)	11	(11.5%)	0.235
Hematologic disease, immunosuppressed	3	(3.1%)	4	(4.2%)	1.000
Solid organ transplant	0		1	(1.0%)	1.000

^a^calculated as weight in kilograms divided by height in meters squared

^b^long term care, rehabilitation clinic and other hospital

^c^obesity is defined as a body mass index greater than 30

### Symptoms

Time from onset of symptoms to hospital admission was longer in the COVID-19 group (median 7 days, IQR 3–10) compared to the influenza group (median 3 days, IQR 2–5, *p* < 0.001). The most commonly reported symptoms were cough (69% vs 76%, *p* = 0.258) and fever (68% vs 65%, *p* = 0.647) in both groups. Symptoms were similar between groups except that COVID-19 patients more frequently reported dyspnea compared to influenza patients (49% vs 32%, *p* = 0.019). On the other hand, coryza (7% vs 13%, *p* = 0.029) and sore throat (2% vs 18%, *p* = 0.006) were less common among COVID-19 compared to influenza patients (Table 2).

**Table 2:** Symptoms reported by COVID-19 vs Influenza patients at time of hospital admission

Symptoms	COVID-19 (*n* = 96)		Influenza (*n* = 96)		*p*-value
Days since symptom onset, median (IQR)^a^	7	(3–10)	3	(2–5)	< 0.001*
General symptoms					
Reduced general condition, malaise	77	(80.2%)	72	(75.0%)	0.387
Fever, chills	65	(67.7%)	62	(64.6%)	0.647
Fatigue	31	(32.3%)	29	(30.2%)	0.755
Pain symptoms					
Headache	30	(31.3%)	41	(42.7%)	0.100
Limb pain, myalgia	18	(18.8%)	24	(25.0%)	0.295
Arthralgia	17	(17.7%)	18	(18.8%)	0.852
Back pain	2	(2.1%)	5	(5.2%)	0.444
Chest pain	10	(10.4%)	19	(19.8%)	0.070
Respiratory symptoms					
Cough	66	(68.8%)	73	(76.0%)	0.258
Dyspnea	47	(49.0%)	31	(32.3%)	0.019*
Sputum	15	(15.6%)	26	(27.1%)	0.053
Coryza, nasal congestion	7	(7.3%)	17	(17.7%)	0.029*
Sore throat	2	(2.1%)	12	(12.5%)	0.006*
Gastrointestinal symptoms					
Dysphagia	0		5	(5.2%)	0.059
Anorexia, weight loss	23	(24.0%)	20	(20.8%)	0.604
Diarrhea	18	(18.8%)	21	(21.9%)	0.590
Nausea, emesis	14	(14.6%)	13	(13.5%)	0.836
Abdominal or stomach pain	7	(7.3%)	7	(7.3%)	1.000
Neurological symptoms					
Dizziness	16	(16.7%)	23	(24.0%)	0.209
Confusion	11	(11.5%)	9	(9.4%)	0.637
Syncope	6	(6.3%)	7	(7.3%)	0.774
Dysarthria, paresthesia	4	(4.2%)	7	(7.3%)	0.352
Somnolence	4	(4.2%)	2	(2.1%)	0.683

^a^Time from first onset of symptoms to admission to the hospital

### Vital signs and clinical scores

COVID-19 patients had a higher systolic blood pressure of 137 mmHg (IQR 120–150 mmHg) compared to influenza patients (median 129 mmHg, IQR 116-138 mmHg, *p* = 0.008). The median oxygen saturation (on room air) was slightly lower in the COVID-19 group (93%, IQR 91–95% versus 94%, IQR 92–97%, *p* = 0.033). CURB-65 and quick-SOFA scores were similar between groups (Table 3).

**Table 3:** Vital signs of COVID-19 vs Influenza patients at time of hospital admission

	n^a^	COVID-19		Influenza		*p*-value
Vital signs						
Systolic blood pressure (mmHg), median (IQR)	95/89	137	(120–150)	129	(116–138)	0.008*
Diastolic blood pressure (mmHg), median (IQR)	95/89	77	(67–85)	74	(66–80)	0.071
Heart rate (beats per minute), median (IQR)	95/89	90	(80–95)	90	(78–104)	0.396
Glasgow coma scale, median (IQR)	95/94	15	(15–15)	15	(15–15)	0.686
No additional oxygen therapy, n (%)	96/96	65	(67.7%)	68	(70.8%)	0.693
Oxygen saturation room air, median (IQR)	93/88	93	(91–95)	94	(92–97)	0.033*
Respiratory rate, median (IQR)	62/28	21.5	(18–25)	23	(17–29)	0.692
Temperature (centigrade), median (IQR)	91/85	37.6	(37–38.2)	37.7	(36.8–38.7)	0.439
Scores						
CURB-65 score, median (IQR)	62/28	1	(0–2)	1	(0–2)	0.208
Quick-SOFA, median (IQR)	62/28	1	(0–1)	1	(0–2)	0.812

^a^data available for n COVID-19/n Influenza patients

### Laboratory findings

**Fig. 3:**
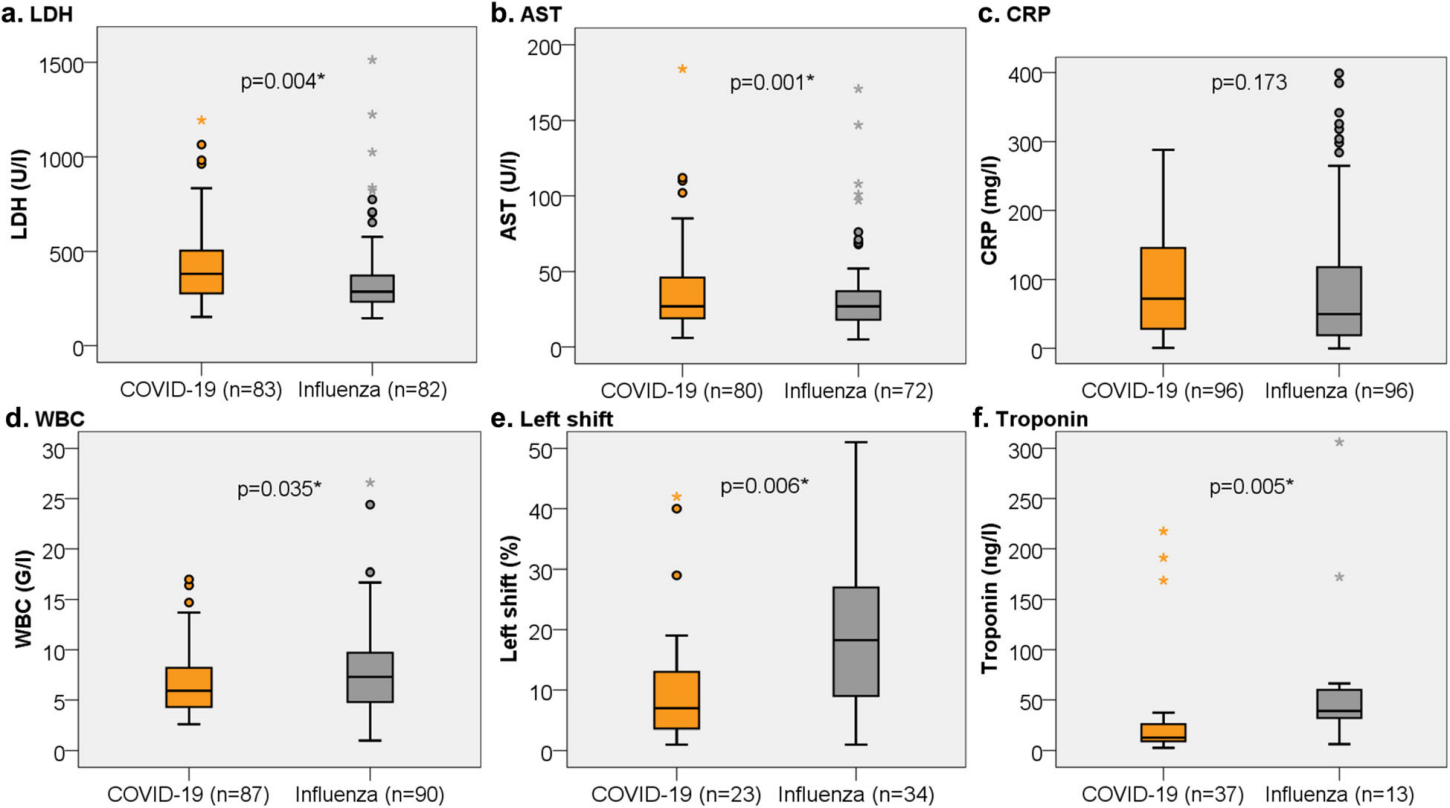
Laboratory results (selected values) by patient group. Note: in the AST graphic (b.) one value (AST 538 U/l) is not displayed in the influenza group. Abbreviations: LDH, lactate dehydrogenase; AST, aspartate aminotransferase; CRP, C-reactive protein; Left shift, left shift of neutrophils; WBC, white blood cell count

COVID-19 patients had a higher median lactate dehydrogenase of 381 U/l (IQR 276–513 U/l) in comparison to 286 U/l (IQR 233–372 U/l, *p* = 0.001) in influenza patients. Furthermore, gamma-glutaminetransferase (median 44 U/l, IQR 28–69 U/l vs 31 U/l, IQR 22–52 U/l, *p* = 0.004) and aspartate transaminase (median 47 U/l, IQR 32.5–70 U/l vs 33 U/l, IQR 22–43 U/l, *p* = 0.001) were higher in COVID-19 compared to influenza patients. Median C-reactive protein (CRP) was similar in both groups (72 mg/l, IQR 28–146 vs 50 mg/l, IQR 19–118). The median white blood cell count was lower in COVID-19 (5.9 G/l, IQR 4.3–8.3G/l) in comparison to influenza patients (7.5 G/l, IQR 4.8–9.9 G/l, *p* = 0.040), and band neutrophils were also less common, with 10% (IQR 5–14%) in the COVID-19 group versus 18% (IQR 9–27%, *p* = 0.006) in the influenza group (Fig. 3).

### Other microbiology results

Blood cultures were positive with relevant pathogens in 2/77 (2%) COVID-19 and in 8/73 (8%) influenza patients (*p* = 0.100). Zero out of 10 (0%) COVID-19 patients and two out of nine (2%) influenza patients had a positive streptococcal urine antigen (*p* = 0.368).

### Recursive partitioning

**Fig. 4:**
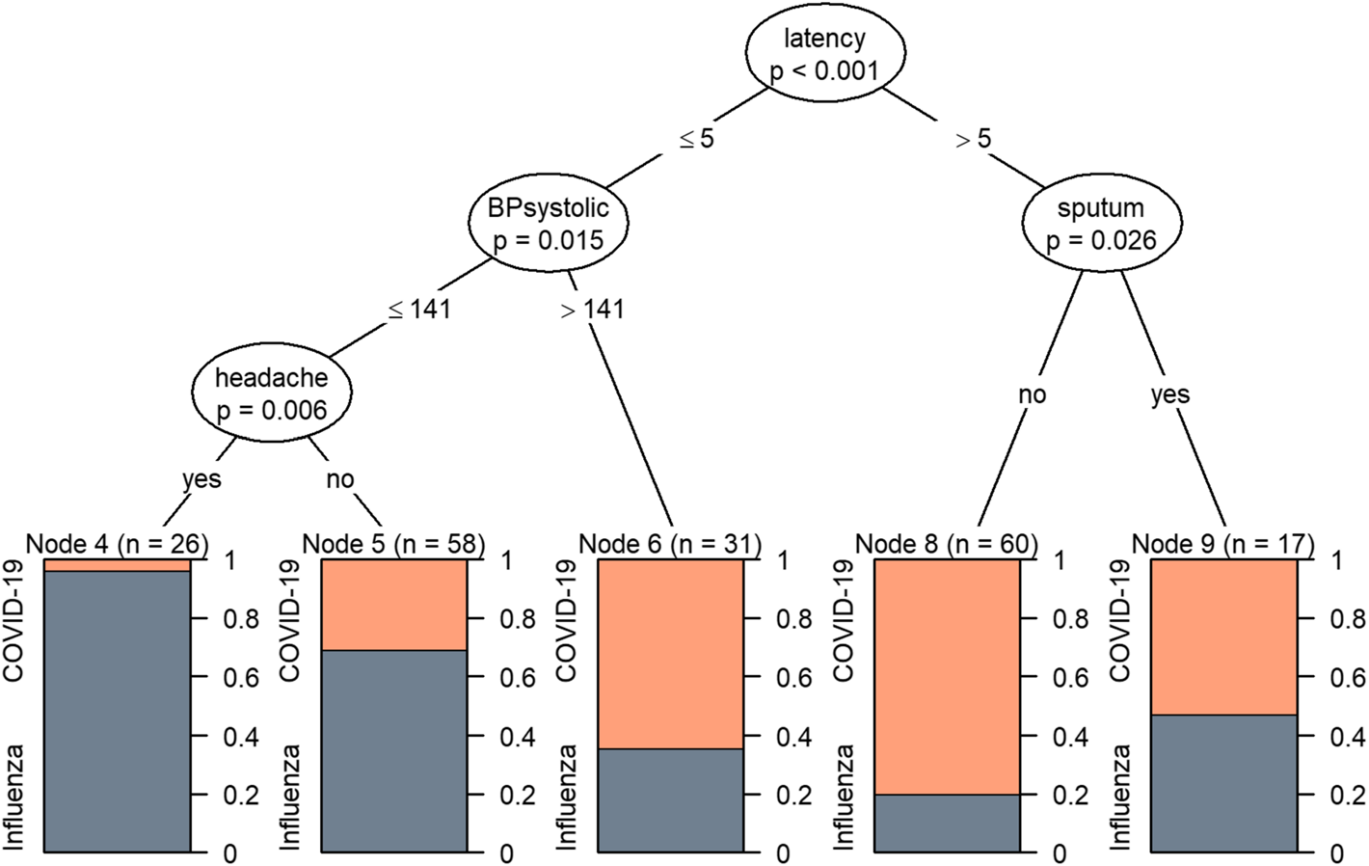
Recursive partitioning tree classifying patients into subsets based on symptoms and vital signs. Proportion of COVID-19 or influenza cases ≥80% in Node 4 and Node 8. Abbreviations: Latency, time from first onset of symptoms to admission to the hospital; BDsystolic, systolic blood pressure in mmHg

The following variables were predictive for COVID-19 in the recursive partitioning tree (Fig. 4): longer time from symptom onset to hospital admission (> 5 days), systolic blood pressure > 141 mmHg, lack of productive sputum, and lack of headache. No laboratory values were identified as relevant predictors in this analysis. Two out of five classification subsets including 86 of 192 patients (45%) were considered as clinically helpful: node 4 (a combination of symptom onset ≤5 days, systolic blood pressure ≤ 141 mmHg, and presence of headache) with 96% of influenza patients and node 8 (combination of symptom onset > 5 days and lack of productive sputum) with 80% of COVID-19 patients (Fig. 4). The three other subsets, including 106 patients (55%), included similar proportions of the two diseases and therefore provided no useful information. In other words, if the associated variable combinations had been used for diagnosis, 73 patients (38%) would have been diagnosed correctly, 13 patients (7%) would have been diagnosed incorrectly, and no diagnosis would have been made for 106 patients (55%).

### Outcome

The proportion of ICU admissions was similar between COVID-19 and influenza patients (19% vs 16%, *p* = 0.566). The median ICU LOS was 4 days (IQR 1.5–15) for COVID-19 and 2 days (IQR 1–8, *p* = 0.138) for influenza patients. However, median hospital LOS was 8 days (IQR 4–12 days) for COVID-19 and 5 days (IQR 3–9 days) for influenza patients (*p* = 0.014). Antibiotic treatment was less (22% vs 46%, *p* < 0.001) and corticosteroids were more frequently (10% vs 1%, *p* = 0.005) administered to COVID-19 patients than to influenza patients. In-hospital mortality was considerably higher in COVID-19 patients in comparison to influenza patients (16% vs 5%, *p* = 0.018).

## Discussion

In this single-center study, we show that the spectrum of clinical presentations overlaps considerably between COVID-19 and influenza patients. However, we identified several variables, which might help to discriminate these patients, the most important being a longer latency between symptom onset and hospital admission for COVID-19 compared to influenza patients. The relatively large sample size is highly representative of all hospitalized patients with these conditions, and the analysis of data from the same time period and the same hospital strengthens the comparability between patient groups.

The variable that discriminated best between COVID-19 and influenza was the time between symptom onset until hospital admission. This finding probably reflects the fact that clinical deterioration commonly occurs on day 5 to 8 after symptom onset in COVID-19 patients, which then causes patients to seek medical care. This important difference between COVID-19 and influenza patients, which has previously been reported by another study [14], could be helpful in the early assessment of patients with ARI. We can however not exclude that public lockdown measures, which were in place during the time when most COVID-19 patients presented to our hospital, may have prolonged the time delay between symptom onset and hospital admission in some patients. Several other variables were significantly different between patient groups. Males were more common among COVID-19 patients. Indeed, male sex is one of the most important risk factors for severe COVID-19 [21]. However, two previous studies could not confirm this association, potentially because of their small sample sizes [12, 13]. Influenza patients had a higher comorbidity score than COVID-19 patients, which corresponds to previous observations [12].

Regarding symptoms, differentiating COVID-19 from influenza patients is challenging. Fever and cough are frequent symptoms in both patient groups. We observed more COVID-19 patients with dyspnea than influenza patients, being in line with the slightly lower oxygen saturation in COVID-19 patients; however, opposite findings have been reported by others [12]. On the other hand, coryza, nasal congestion and sore throat were less common among COVID-19 patients, which has been found in previous studies [12, 14]. Sputum production has been reported to occur more frequently in influenza patients [12, 14]; accordingly, productive sputum was identified as important discriminating variable in the classification tree. Headache was slightly more common in influenza (43%) compared to COVID-19 patients (31%). In the literature, headache has been reported in about 14% of COVID-19 patients [22], while the frequency seems indeed to be higher in influenza patients, with some studies reporting a prevalence of over 50% [23]. A limitation of our study is the fact that anosmia or loss of taste were not routinely asked in this early phase of the pandemic. Other studies show that these symptoms might be very useful in distinguishing COVID-19 from other respiratory illnesses, including influenza [12, 14].

Systolic blood pressure was higher among COVID-19 patients. This could be due to the fact that arterial hypertension is one of the most important risk factors for severe COVID-19 [21]. Other studies reported no difference in the systolic blood pressure values [13]. We identified several laboratory values which were different in COVID-19 and influenza patients. These included the lactate dehydrogenase and the aspartate transaminase (both higher in COVID-19 patients). However, these variables were not helpful in the generation of the classification trees, potentially because of the overlapping range of values between the two groups. Furthermore, these two laboratory findings are generally not very specific. We observed a lower white blood cell count in COVID-19 patients which has been reported by others [10, 14]. Also, band neutrophils were more common in influenza patients, supporting the hypothesis of more frequent bacterial superinfection, as previously suggested [24].

This is the first study to present a clinical classification tree to discriminate COVID-19 from influenza patients. This method has the advantage that it easily identifies interaction effects between variables, which are not readily captured with traditional multivariable analyses. As seen in our tree, different variables are important in discriminating patient groups, depending on the time since symptom onset. For instance, sputum production as a sign for bacterial superinfection plays a role after a latency of at least 5 days after symptom onset, but seems to be of minor importance shortly after patients become symptomatic. We were able to classify almost half of patients with reasonable certainty in one of either group, using parameters which can be simply collected in any healthcare setting. However, external validation of these data is necessary.

COVID-19 patients fared worse than influenza patients. Not only was the risk of complication higher, they also had a longer hospital LOS and considerably higher in-hospital mortality. These findings have been reported by others and support the current notion of COVID-19 being a more hazardous condition than seasonal influenza, at least for hospitalized patients [14, 25].

Our study has several limitations in addition to the ones mentioned above. First, due to the retrospective study design patient symptoms were not systematically collected, which could have introduced reporting bias. Second, the exploratory character of our analysis comes along with the possibility of type I errors, finding statistically significant associations where there are in fact none. Third, clinical presentation of seasonal influenza may vary from year to year due to differences in circulating subtypes. The same might also apply to the clinical presentation of COVID-19, in particular with the emergence of B.1.1.7 and other viral variants.

## Conclusions

Finally, distinguishing COVID-19 from influenza patients at time of hospital presentation poses a clinical challenge. Time from symptom onset to hospital admission is a key variable which can be used for this purpose. Furthermore, we propose an easy-to-use classification tree, which is able to provide helpful estimates regarding COVID-19 or influenza diagnosis for a considerable proportion of patients at time of hospital admission. Prospective studies with systematic data collection are however needed to confirm these findings.

## Supplementary Information

**Additional file 1.**

## Data Availability

The datasets from this study are available from the corresponding author on reasonable request.

## Abbreviations

ARDS: Acute Respiratory Distress Syndrome
ARI: Acute Respiratory Illness
CCI: Charlson Comorbidity Index
COVID-19: Coronavirus Disease 2019
CRF: Case Report Form
CRP: C-reactive Protein
ICU: Intensive Care Unit
IQR: Interquartile Range
PCR: Polymerase Chain Reaction
SARS-CoV-2: Severe Acute Respiratory Syndrome Coronavirus 2
